# Microbial communities developing within bulk sediments under fish carcasses on a tidal flat

**DOI:** 10.1371/journal.pone.0247220

**Published:** 2021-02-25

**Authors:** Yasutake Kawamoto, Hiromi Kato, Yuji Nagata, Jotaro Urabe

**Affiliations:** Graduate School of Life Sciences, Tohoku University, Sendai, Miyagi, Japan; University of Illinois at Urbana-Champaign, UNITED STATES

## Abstract

Animal carcasses are often brought into tidal flats where they are at the boundary between terrestrial and marine ecosystems. Since these carcasses act as microhabitats with large amounts of energy and nutrients, they likely develop unique bacterial assemblages in the ambient sediment, which in turn may stimulate colonization of other organisms such as protozoans. However, little is known about the microbial assemblages colonized in sediment around animal carcasses in the tidal zone. Herein we examined the bacterial and ciliophoran assemblages developed in association with fish carcasses by incubating the carcasses in the Higashiyachi tidal flat (Sendai, Japan). We collected sediment samples at 2, 9, and 42 days of incubation and analyzed the bacterial and ciliophoran assemblages by 16S and 18S rRNA gene amplicon sequencing. We observed significant differences in the composition and relative abundance of bacterial and ciliophoran operational taxonomic units (OTUs) between the sediments with and without the carcasses. Our analyses suggest that these unique assemblages were created through the direct effects of the carcass and indirect effects through interactions between bacteria and ciliophorans. These results also suggest that animal carcasses developed a temporally unique microbial food web in the sediments close to the carcasses, although it disappeared for several weeks.

## Introduction

Animal carcasses are omnipresent and play functionally important roles for material cycling and thus sustaining ecosystems [1–4]. However, carcasses disappear rather quickly due to their consumption by scavengers and decomposition by bacteria [5, 6]. For bacteria, carcasses provide large amounts of energy and nutrients, although these are ephemeral. Several studies have shown that bacterial assemblages developed in the vicinity of carcasses differ significantly from those in the environments without such carcasses [7–9]. However, it is not clear whether the bacterial assemblages developed in the vicinity of carcasses are composed mainly of bacteria inhabiting the surrounding environment or unique bacteria specific to the carcasses.

An increase in bacterial abundance associated with a carcass provides diet resources for bacterivores such as Ciliophorans. In addition, an increase in bacterial activities results in the consumption of oxygen, thus creating an anoxic condition in ambient environments. Accordingly, carcasses may allow the colonization of phylogenetically specific organisms that prey on bacteria and resist low oxygen conditions. However, little is known about the bacterivores that are colonized in association with bacterial assemblages that develop in the vicinity of carcasses.

Tidal flats form the boundary between terrestrial and marine ecosystems. Therefore, various organic matter, including carcasses, are brought into tidal flats by inflows and drifts from both ecosystems. However, animal carcasses such as fish ones are rarely found in tidal flats probably because they are rapidly decomposed [5], flowed away at storms, or covered by sands and silts and thus buried in the sediment. Accordingly, few studies have examined the microbial communities occurring in association with carcasses in tidal flats. In addition, due to the lack of suitable methods for examining microbial organisms (e.g. [10, 11]), it has been difficult to examine the taxonomic composition of microbial communities developed in association with these carcasses.

Therefore, in this study, we examined microbial communities that had developed in association with fish carcasses at a tidal flat by performing 16S and 18S rRNA amplicon sequencing. We used sardines as model animals of carcasses since large numbers of dead sardines often land on the coasts (https://japantoday.com/category/national/tons-of-dead-sardines-wash-up-on-chiba-shore). In addition to bacteria, we examined Ciliophorans since they are important bacterivores in aquatic ecosystems [12, 13]. We tested the following three hypotheses: (1) bacterial assemblages developed in the sediment close to a fish carcass are composed mainly of taxa in the ambient environments, (2) but these taxa’s relative abundances differ largely between the sediment vicinity of fish carcass and that a little away from the carcass, and (3) fish carcasses also modify the environmental condition in the surrounding sediments so that phylogenetically specific Ciliophora colonize through interactions with the bacterial assemblages. The test of these hypotheses would contribute to understanding the structural and functional roles of the microbial communities in decomposing carcasses in aquatic ecosystems.

## Materials and methods

### Study site and experimental set-up

This study was performed at the Higashiyachi tidal flat (38° 11.4’ N, 140° 57.7’ E), located at the coast of northeastern Japan and faced the Pacific Ocean. Because the intertidal flat was not privately owned or legislatively protected, no special permits were required to perform field experiments in the area. In this study, sardines (*Sardinops melanostictus* body size ca. 20 cm) purchased in a fish market were used as model organisms of carcasses. No protected species were involved in this study. To initiate the experiment, each single dead fish was put in a 500-mL square plastic bottle (72×72×140 mm3) with 28 1-cm holes distributed on all of the bottle’s sidewalls. The bottle was then filled with tidal flat sediments that had been passed through a 1-mm mesh net for the removal of macro-organisms. We created a total of nine such bottles with a fish carcass and used these as the fish-treatment (hereafter referred to as the "F-treatment") bottles. We also prepared 15 bottles filled with the tidal flat sediment without a fish carcass and used these for the near-fish treatment (N-treatment; buried close to the F-treatment bottles) and control treatment bottles (C-treatment; buried distant from all other bottles).

To initiate the experiment, on 8 June 2016, we buried nine pairs of F- and N-treatment bottles 12 cm deep in the sediment at the Higashiyachi tidal flat, at 3×3 lattice points with 5-m intervals between the points. The N-treatment bottles were placed 10 cm distant from the F-treatment bottles. The C-treatment bottles were also buried 12 cm deep in the sediments but ≥5 m distant from any other bottles.

At 2, 9, and 42 days later, we collected 2 C-treatment bottles and 3 bottles from each F- and N-treatments. We placed these bottles in a cool container, took the containers to the laboratory and sampled the bulk sediments in the bottles. In F-treatments, we carefully sampled not to contain fish carcasses in the bottle on day 2. However, on days 9 and 42, we could not separately sample the sediments apart from fish carcasses since the carcasses were progressively decomposed and already mixed with the sediments. These bulk sediment samples were homogenized and stored at −80°C until the DNA analyses.

### DNA analyses

In each sample, 0.25 g of sediment was subsampled and used to extract total DNA with a PowerSoil^®^ DNA isolation kit (MoBio Laboratories, Carlsbad, CA). From each sediment sample, we obtained ≤100 μL of DNA solution. We used the V3 to V4 regions of the 16S rRNA gene for prokaryotes and the V4 region of the 18S rRNA gene for Ciliophora as the taxonomic marker genes.

The polymerase chain reaction (PCR) amplification of 16S rRNA genes was performed in 50 μL of 1× *Ex Taq* buffer (2 mM Mg2+ Plus; TaKaRa Bio, Kusatsu, Japan) containing 0.2 mM dNTPs, 1.25 U of *TaKaRa Ex Taq*^™^ (TaKaRa Bio), 0.2 μM each of the forward and reverse primers, and 10 μL of template DNA. To avoid the amplification of eukaryotic rRNA genes, we used 342F-806R primers [14] with overhang adapter sequences for compatibility with the Illumina index and sequencing adapters. The PCR procedure was conducted as follows: 95°C for 30 sec, followed by 25 cycles consisting of 95°C for 30 sec, 52°C for 30 sec, and 72°C for 45 sec, and then a final cycle of 72°C for 10 min. According to the manufacturer’s protocols, the amplicons were sequenced (300 bases, paired-end) on the Illumina MiSeq platform (Illumina, San Diego, CA).

For ciliophorans, 18S rRNA genes were amplified by a nested PCR. The 1st PCR amplification with the ciliophoran-specific primers CilF, CilR I, CilR II, and CilR III [15] was performed in 30 μL of the mixture mentioned above with two times the amount of the polymerase. The appropriate amount of reverse primers calculated by the total quantity was included. The PCR procedure was conducted as follows: 95°C for 5 min, followed by 35 cycles consisting of 94°C for 45 sec, 58°C for 1 min, and 72°C for 1 min, and then a final cycle of 72°C for 10 min. Subsequently, TAReuk454FWD1 and TAReukREV3, which are eukaryote-specific primers, were adopted for a 2nd PCR to amplify the hyper-variable V4 region, which is composed of about 480 bp [16]. The overhang adapters were also attached to these primers. The PCR was performed in 50 μL of the same mixture as used for the 1st PCR, except that 0.5 μL of the template amplicon DNA was used in place of 10 μL of template DNA; some thin amplicons were added (5 μL). The PCR program consisted of a single cycle of 95°C for 5 min; followed by 10 cycles of 94°C for 30 sec, 57°C for 45 sec, and 72°C for 1 min; then 25 cycles at a low annealing temperature at 49°C; with a final cycle at 72°C for 2 min. The nested PCR amplicons were sequenced in the same way as the amplicons of the 16S rRNA gene.

The sequence data were processed by USEARCH v10.0.240 [17]. Paired-end reads were assigned to each sample based on their unique barcode. We merged the reads based on overlapping regions within paired-end reads. According to Illumina’s protocol, we checked if the reads from the PhiX control library, which was added as a spike, were already removed automatically by the Miseq platform. We truncated the primer sequence from the merged sequence by using TagCleaner 0.16 [18]. After the initial trimming, we discarded singleton reads with USEARCH v10.0.240 to minimize potential errors [19]. We applied a sequence similarity of 97% [20] to delineate taxa as operational taxonomic units (OTUs) and annotated them for both bacteria and ciliophorans using the amplicon analysis pipeline of the SILVA project (SILVAngs 1.4) [21]. Chimera check was run to the output OTU sequences with UCHIME2 [22] using SSU rRNA database as references: the SILVA database (SSU r138) was used for bacteria and PR2 v4.7.2 [23] for ciliophorans. We assigned an OTU sequence to each read with VSEARCH v2.15.0 [24]. For the analysis of ciliophorans, we removed non-specific OTUs such as dinoflagellates or Apicomplexan protozoans. To compare samples with different numbers of reads, sample reads were rarefied according to the coverage-based rarefaction method [25] before performing the statistical analyses below.

### Statistical analyses

In the subsequent analyses, OTUs were used as the units of taxa. For both bacteria and ciliophorans, we examined the similarity in taxon composition among the treatments using the Horn index [26], a simplified version of the Morisita index, since this index can avoid the effect of the sample size on any estimates. To estimate the Horn index, we first calculated the relative abundance of each OTU using the number of reads for the given taxon against total reads in the sample. Note that this index is very sensitive to the relative abundance of dominant species, and it tends to underestimate similarity if many common taxa with low abundance are present. To avoid such an underestimation, we estimated the index after performing a log-transformation of the abundance data [27]. Using the Horn index, we performed a non-metric multidimensional scaling (nMDS) analysis to visualize the degree of similarity among samples within and between treatments. Significant differences in the similarity index between the treatments were tested using a pairwise permutational multivariate analysis of variance (PERMANOVA) with 4,999 permutations in the R package *vegan* [28].

We estimated the nestedness-resultant dissimilarity (β_nes_) [29] to examine if the OTU composition of a microbial assemblage was parts of those of other microbial assemblages. We also estimated the Simpson pairwise dissimilarity (β_sim_) [30], which discriminates the contribution of species turnover to dissimilarity from the contribution of nestedness. To determine whether the ciliophorans that developed in treatment were derived from phylogenetically limited or various groups, we estimated the mean pairwise phylogenetic diversity (MPD), according to Webb et al. [31]. In MPD, a small value means that the ciliate assemblage is composed of taxa from phylogenetically limited groups; a large value means that the ciliate assemblage is composed of phylogenetically diverse groups [32]. To calculate the MPD in each sample, we generated a phylogenetic tree of ciliophorans using18S rRNA sequences that were aligned with ClustalW and edited manually with MEGA X [33]. Nucleotide positions that were covered by <65% of aligned sequences were eliminated from the analysis. The phylogenetic tree was constructed with the Minimum Evolution method [34]. The tree reliability was examined by 10,000 bootstraps. The phylogenetic distance between Ciliophora taxa was calculated from the tree. We then estimated the MPD of each sample by using the R package *picante* [35]. The effects of the treatments and the sampling date on the MPD were examined by a two-way ANOVA. We did not perform an MPD analysis for bacterial assemblages in this study since we could not construct reliable phylogenetic trees of the observed bacterial taxa.

A multiple regression analysis on distance matrices (MRM) [36] was performed to examine the effects of the sampling date, fish treatments, and the similarity of ciliophorans on the similarity of the bacterial assemblages. We used the Horn index for the distance matrices of the bacteria and ciliophoran assemblages. In this analysis, we used binary data for the sampling date and fish treatments, which we set as 0 when we compared assemblages from the same sampling date or fish treatments and set as 1 when we compared these between different sampling dates or fish treatments. The significance of the explanatory variables was examined by a randomization test with 10,000 permutations with the α-level at 0.05. The same MRM was also done using ciliate assemblages as the response variable and the Horn index of the bacterial assemblages as an explanatory variable. These MRMs were carried out using the R package *ecodist* [37]. With the full model of MRM, variation partitioning was performed using coefficients of determination (r2) for models with and without each of the explanatory variables according to the procedure described by Legendre and Legendre [38].

### Nucleotide sequence accession numbers

The raw reads of the 16S and 18S rRNA genes sequenced in this study have been deposited in the DDBJ under the accession numbers DRA008552.

## Results

### The state of the carcasses during incubation

**Fig 1:**
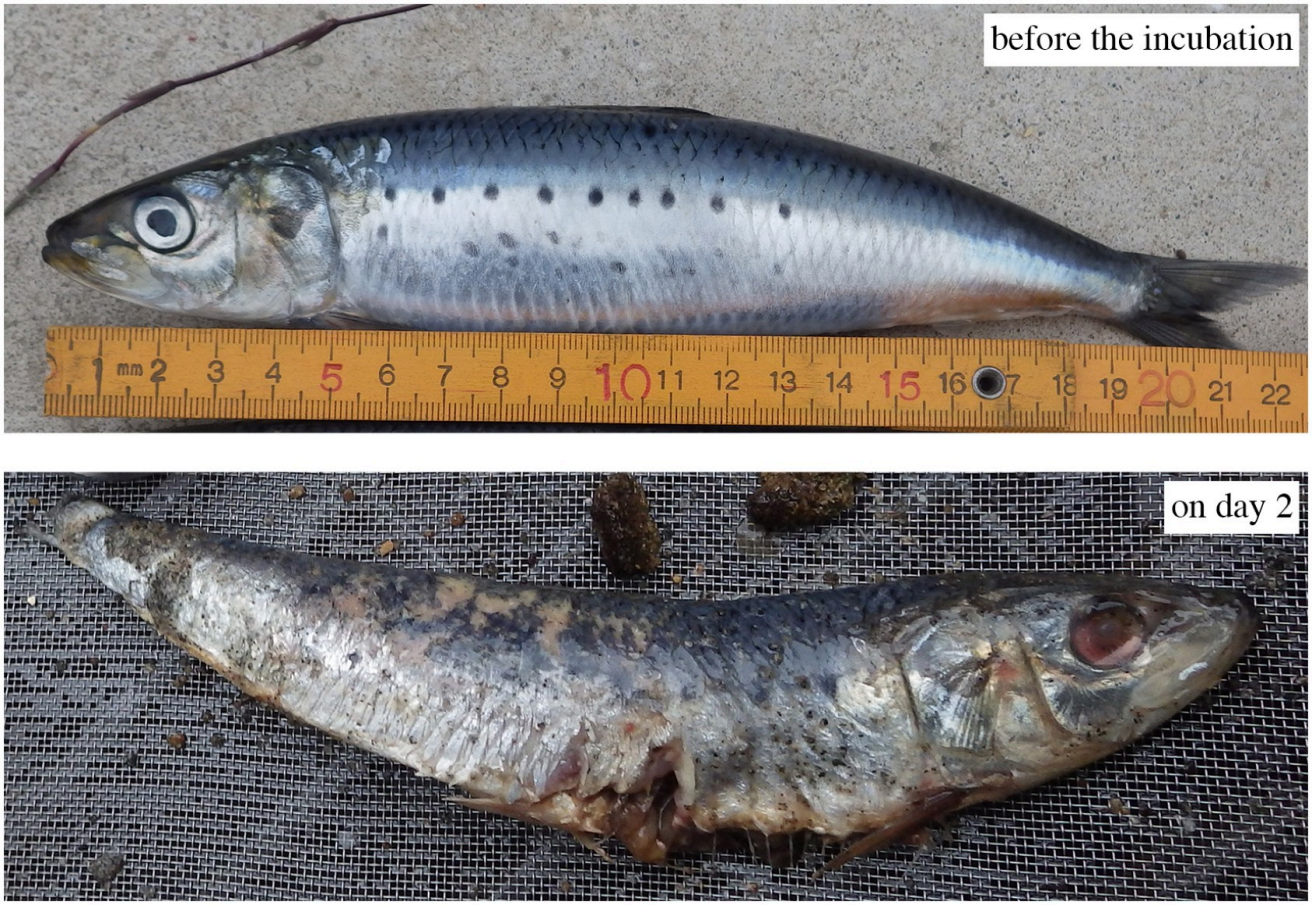
Photographs of *Sardinops melanostictus* before the experiment (upper panel) and at the two days after the incubation (lower panel).

At two days of incubation, the guts and caudal fin of the fish carcasses had started to decay (Fig 1). The scales and skin were peeled off, but the fat layer with the flesh meets was still observed. Sediments in the incubation bottles had become partially darkened. On the 9th day, the carcasses emitted a strong odor that was the strongest during the experiment. The carcasses were fragmented, with slightly bloody coloring on the surface. The sediments in the bottles were much more darkened. On the 42nd day, the smell had weakened, and no fragments of the carcasses were observed. However, unlike the treatments without a fish carcass, the sediments in the incubation bottles were still dark-colored.

### Bacterial assemblages

The amplicon sequencing with the rarefaction procedure recorded a total of 11,333 OTUs with 2,145,919 reads (S1 Table). In the F-treatment bottles, the number of OTUs ranged from 611 to 2,213 per sample depending on the sampling date and was especially low on day 9 (S1 Table). In the N- and C-treatments, the number of OTUs ranged from 5,124 to 6,849, respectively, and was less temporally changed compare to the F-treatment.

**Fig 2:**
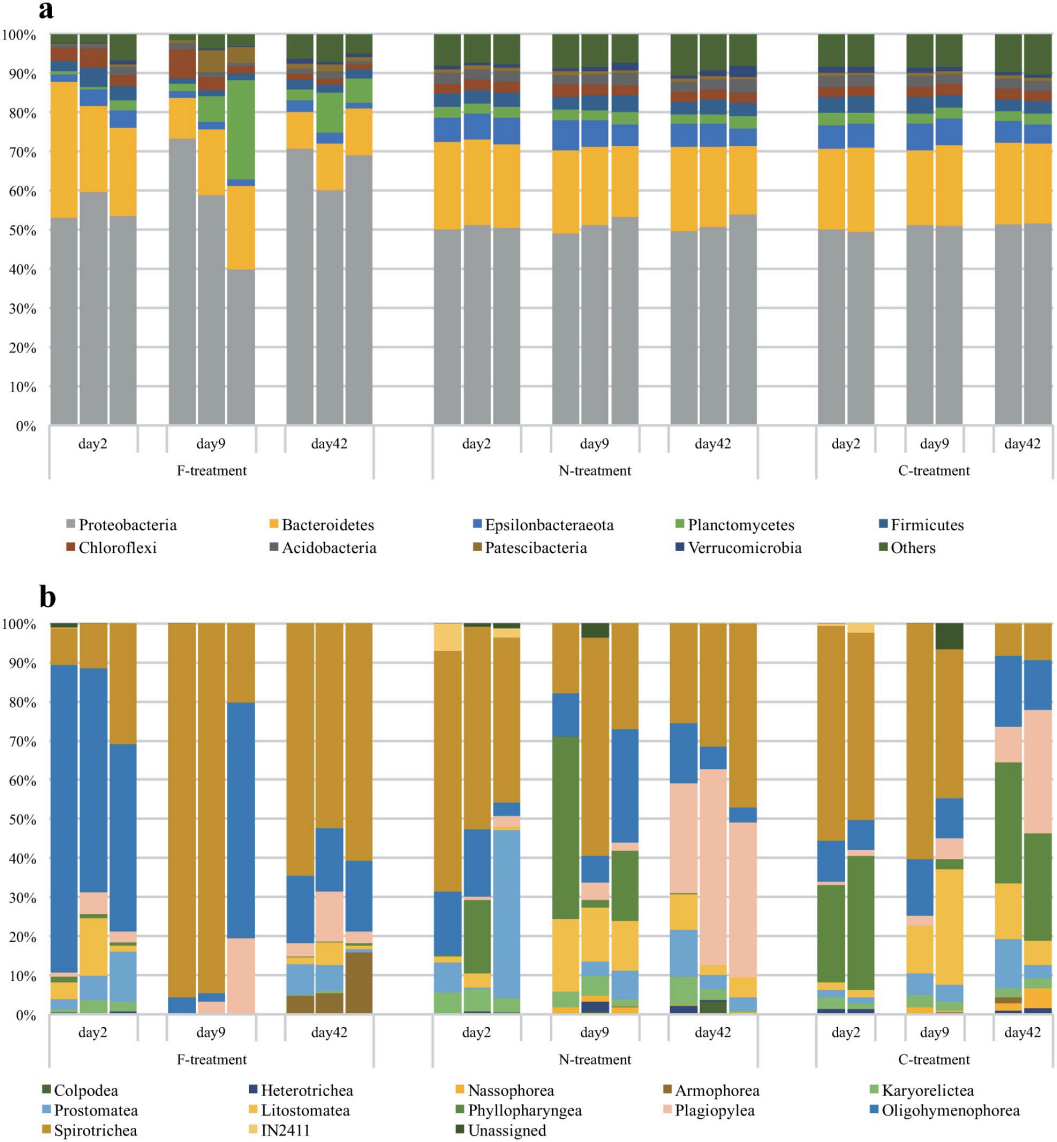
Temporal changes in the microbial assemblages in F-, N- and C-treatments. The relative abundances of (a) different Bacteria phyla based on 16S rRNA sequencing and (b) different Ciliophora classes based on 18S rRNA sequencing.

**Fig 3:**
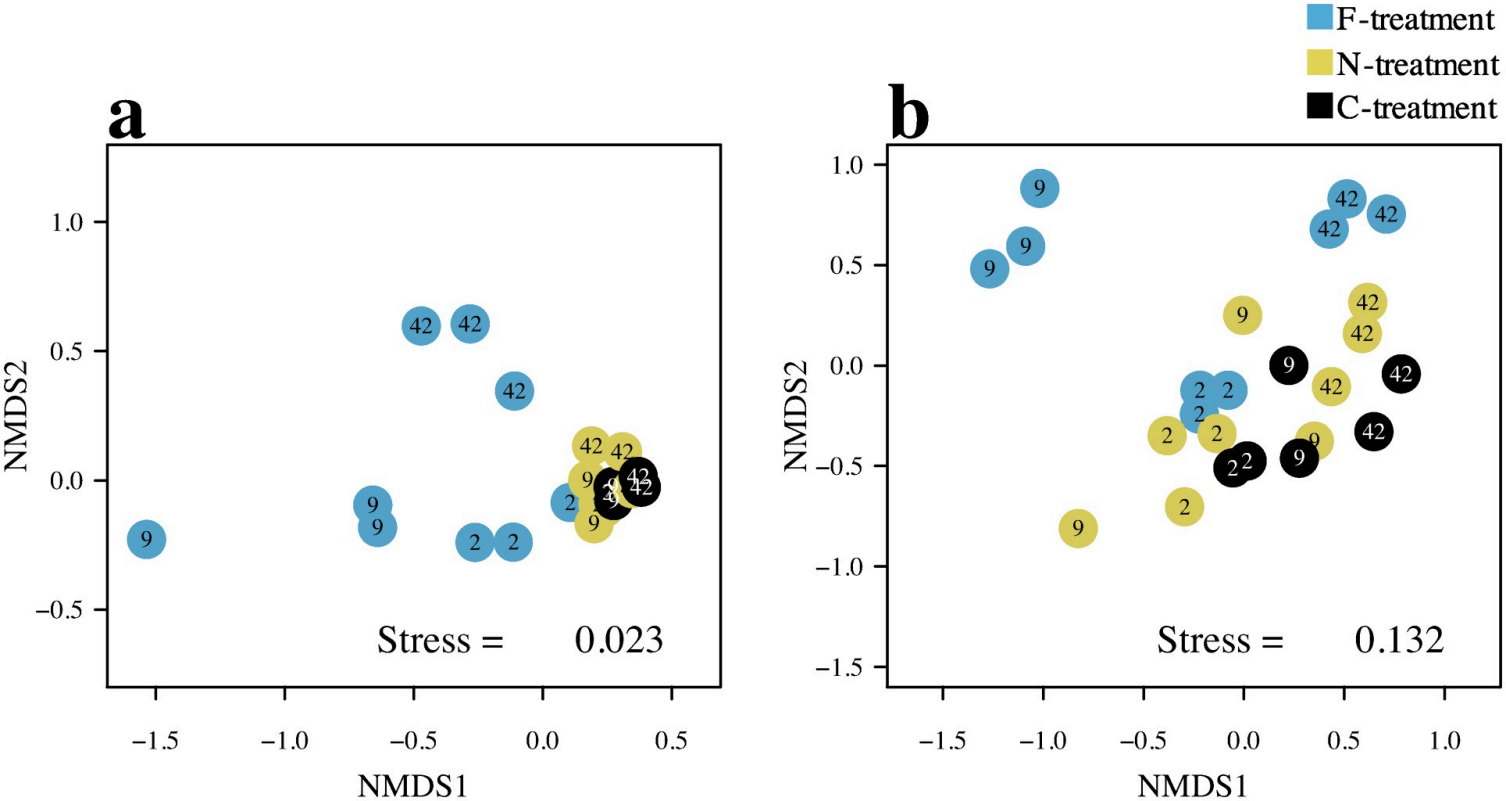
Temporal changes in the similarity among microbial assemblages. The similarities of (a) bacterial and (b) ciliophoran assemblages are shown as non-metric multidimensional scaling plots using the Horn index. Dates examined are denoted by figures in circles.

The taxonomic composition of the bacterial communities at the OTU level varied widely among the treatments but was much less varied among the samples in the same treatments (S2 Table). Such variations can be found even at the phylum level (Fig 2a). According to the PERMANOVA with Bonferroni corrections (Table 1), the OTU composition of the bacterial assemblages was significantly different between the F- and C-treatments and between the F- and N-treatments but not between the N- and F-treatments. The pattern of temporal changes in the OTU composition also differed between the F-treatment and the other two treatments (Fig 3a).

**Table 1:** Results of pairwise PERMANOVAs for examining the effects of treatments on bacteria and ciliophoran assemblages.

Source		d.f.	SS	MS	Pseudo F	Adjusted p
Bacteria	F vs. N treatments	1	0.53669	0.53669	9.636	<0.01
	Residuals	16	0.89115	0.0557		
	Total	17	1.42784	1		
	F vs. C treatments	1	0.52773	0.52773	8.1528	<0.01
	Residuals	13	0.84149	0.06473		
	Total	14	1.36923	1		
	N vs. C treatments	1	0.013612	0.0136122	1.6289	n. s.
	Residuals	13	0.108634	0.0083564		
	Total	14	0.122246	1		
Ciliophorans	F vs. N treatments	1	0.4328	0.43281	1.6839	n. s.
	Residuals	16	4.1125	0.25703		
	Total	17	4.5453	1		
	F vs. C treatments	1	0.6314	0.63138	2.6478	<0.05
	Residuals	13	3.1	0.23846		
	Total	14	3.7314	1		
	N vs. C treatments	1	0.22503	0.22503	1.0344	n. s.
	Residuals	13	2.82799	0.21754		
	Total	14	3.05301	1		

In the bacterial assemblages, *Proteobacteria* were the most abundant phylum, followed by *Bacteroidetes* in all three treatments. *Planctomycetes* were more abundant on days 9 and 42 and less abundant on day 2 in the F-treatment compared to the N- and the C- treatments. In the former treatment, *Chloroflexi* appeared abundantly on days 2 and 9, and *Patescibacteria* were also abundant on day 9. However, these phyla were less abundant in the N- and C-treatments where *Epsilonbacteraeota*, *Planctomycetes*, *Firmicutes*, *Chloroflexi* and *Acidobacteria* appeared abundantly.

**Fig 4:**
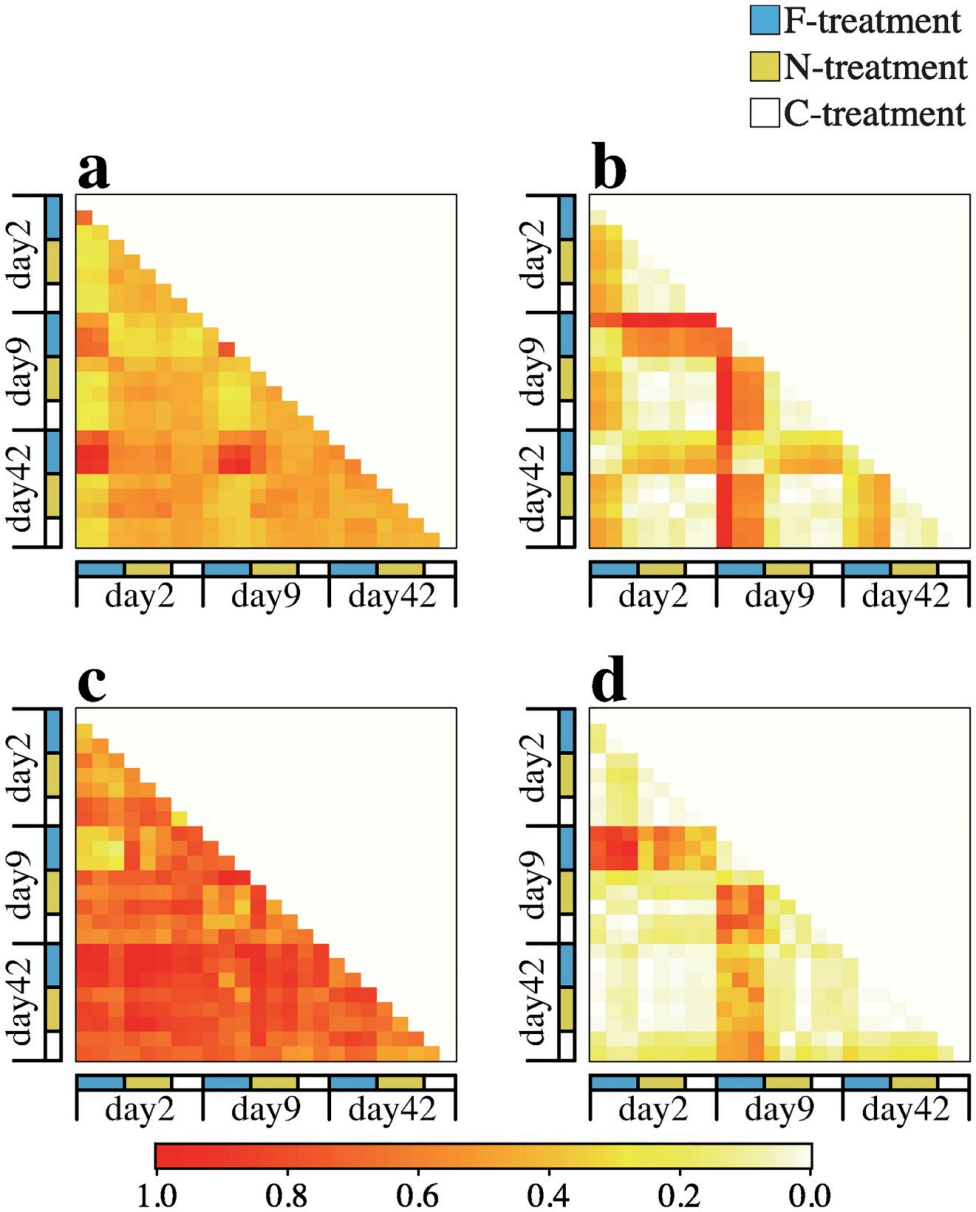
Heatmaps showing the Simpson pairwise dissimilarity (β_sim_) and the nestedness-resultant dissimilarity (β_nes_) indices among the microbial assemblages. (a) β_sim_ and (b) β_nes_ among the bacterial assemblages and (c) β_sim_ and (d) β_nes_ among the ciliophoran assemblages.

To determine whether bacteria communities in the F-treatments were subsets of those in the N- or C-treatments, we examined the β_sim_ and β_nes_ values among samples of the same and different treatments and visualized the relations by creating heat maps (Fig 4a and 4b). In most cases, the β_nes_ values were < 0.1 and β_sim_ values were < 0.3 among the N- and C-treatments samples, indicating that the OTU compositions were relatively similar and contained few nested structures among these samples. However, some of the β_sim_ values among the F-treatment samples collected on different dates were high, indicating that at least a part of the OTUs was temporally turned over. The β_nes_ values between the F-treatment samples and the N- and C-treatment samples were generally >0.2, indicating the existence of a somewhat nested structure between these samples.

### Ciliophora assemblages

The meta-barcoding analysis with the rarefaction procedure recorded 778 OTUs of Ciliophora with 2,597,949 reads in the total samples. The number of OTUs ranged from 85 to 263 per sample, except in F-treatment samples collected on day 9, when only 25 OTUs of Ciliophora were detected (S1 Table).

The OTU composition of the ciliophoran assemblages was similar among the samples within the same treatments (S3 Table), as was observed for the bacterial assemblages. However, compared to the bacterial assemblages, the taxonomic composition of the ciliophorans was less similar between the N- and C-treatments even at the Class level (Fig 3b). For example, Phyllopharyngea occurred abundantly on days 2 and 42 in the control treatment but day 9 in the N-treatment. In the F-treatment, Oligohymenophorea predominately appeared, especially on day 2, although Spirotrichea occurred abundantly on other dates as in some samples of the C- and N- treatments. In the F-treatment, Armophorea, which were rarely observed in the N- and C-treatments, were often found on day 42. Like the bacterial assemblages, the OTU composition of the ciliophorans in the F-treatment differed somewhat from those in other treatments.

According to the PERMANOVA with Bonferroni correction (Table 1), the OTU compositions of the ciliophorans differed significantly between the F- and C-treatment samples but not between the N- and C-treatment samples. In addition, unlike the bacterial assemblages, the OTU composition of the ciliophorans did not differ significantly between the F- and N-treatments, although samples of the latter treatment contained a number of OTUs that occurred in the C-treatment.

**Fig 5:**
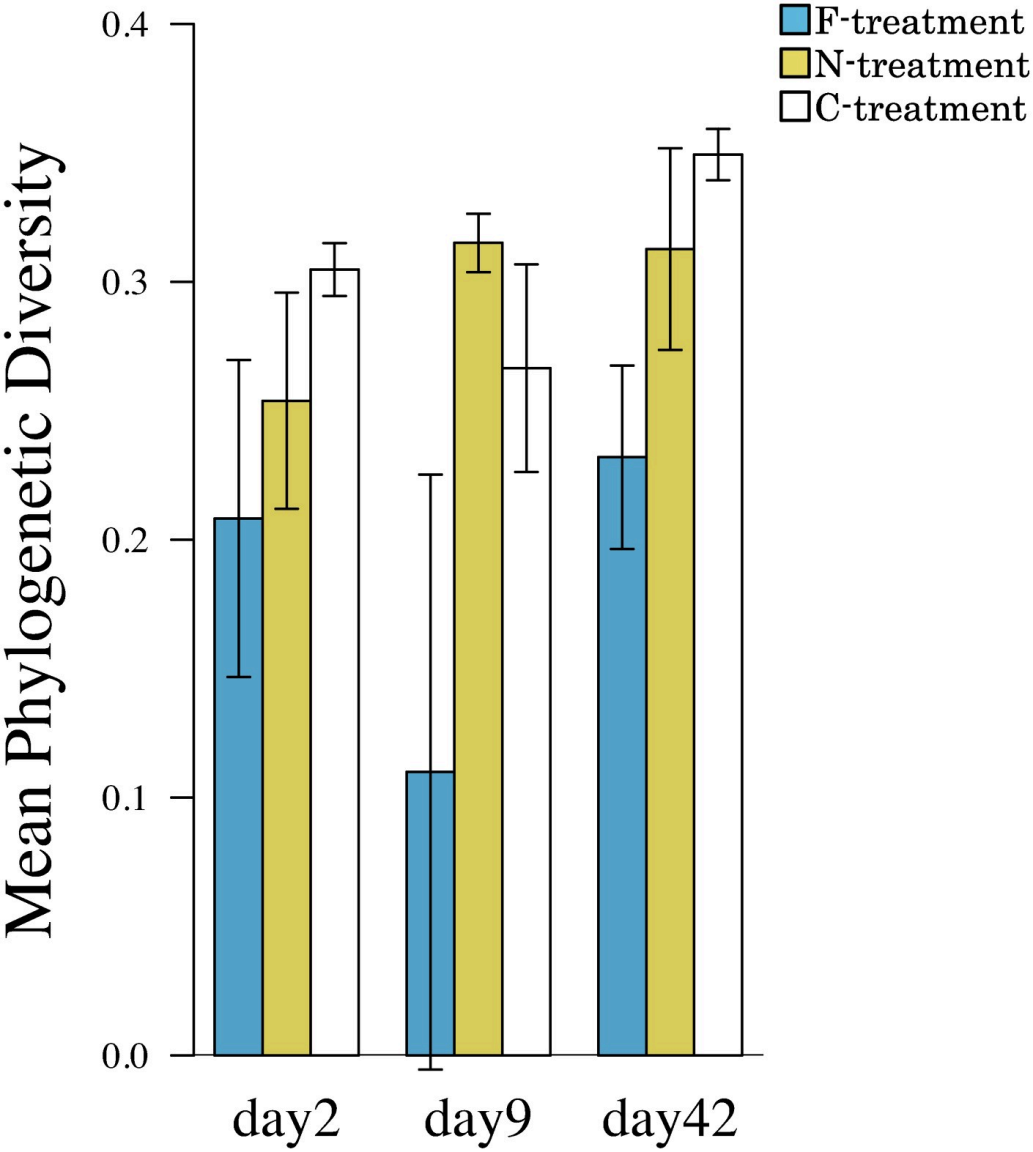
The Mean Phylogenetic Diversity (MPD) of ciliophoran assemblages in each of the three treatments. The vertical bar is the SD on the mean.

In the ciliophoran assemblages, most of the β_sim_ values were >0.4 among the samples regardless of the treatments and sampling date (Fig 4c), indicating that the OTU composition temporally and spatially varied much more compared to the bacterial assemblage. Most of the βnes values were < 0.2 among the samples. The exceptions were the values between the F-treatment samples and other samples collected on day 9 (Fig 4d), indicating that the OTU compositions of the F-treatment samples were a part of those of other samples on this date. To determine whether the ciliophoran assemblages were composed of taxa in phylogenetically specific groups, we estimated the mean pairwise phylogenetic diversity (MPD). According to the two-way ANOVA, the MPD was significantly affected by the treatment (Table 2). Indeed, the estimated MPD was lower in the F-treatment on any date compared to other treatments (Fig 5).

**Table 2:** Results of two-way ANOVAs for examining the effects of sampling date and treatments and their interaction on the mean phylogenetic diversity of a ciliate assemblage.

Variables	d.f.	SS	MS	F	p
Date	2	0.01768	0.00884	2.925	n. s.
Treatment	2	0.07594	0.03797	12.567	<0.001
Date × treatment	4	0.02156	0.00539	1.784	n. s.
Residuals	15	0.04533	0.00302		

### Factors affecting the microbial assemblages

To examine the factors affecting the OTU compositions of the bacteria and ciliophoran assemblages, we analyzed the distance of OTU similarities among samples with commonalities of environmental conditions by performing a multiple regression analysis on distance matrices (the MRM analysis). This analysis showed that the similarity distance of the OTU composition among the ciliophoran assemblages was significantly related to the commonality of the sampling date, the presence/absence of a fish carcass, and the similarity distance of the bacterial OTU composition (S4 Table). Similarly, the similarity distance of the OTU composition among the bacterial assemblages was significantly related to the presence/absence of a fish carcass and the similarity distance of the ciliophoran OTU composition (S4 Table).

**Fig 6:**
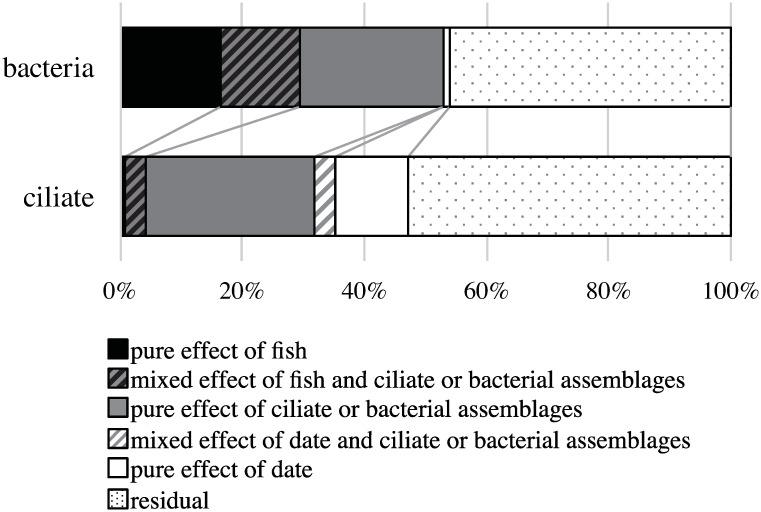
The results of variation partitioning in the Horn indices of microbial assemblages. The Horn indices of bacterial assemblages are partitioned by the sampling date, fish carcass and ciliophoran assemblages, and those of the ciliophoran assemblages by the sampling date, fish carcass and bacterial assemblages.

The variation partitioning showed that 29% of the variation in the similarity of OTU composition in the bacterial assemblages was explained by the presence/absence of a fish carcass, and 36% was explained by the difference in ciliate assemblages (Fig 6). The sampling date explained only 1% of the variation. In the ciliophoran assemblages, only 4% of the variation in the similarity of OTU composition was explained by the fish carcass, and 34% was explained by the difference in bacterial assemblages. The sampling date explained 16% of the variation in the similarity distance of the ciliate OTU composition.

## Discussion

In previous studies, the ecological functions of carcasses of aquatic animals such as salmon [39, 40] and whales [41–43] have been examined, but these studies focused on the carcasses as a dietary source for vertebrate and invertebrate scavenging animals. Although microbial organisms may compete with these scavenging animals for the carbon and nutrients within carrion [4, 44], few studies have examined microbial communities on aquatic animal carcasses (e.g., [43, 45]), and no studies have examined the structure and dynamics of microbial communities that developed in association with animal carcass, especially in aquatic ecosystems. This study suggested that fish carcasses developed a unique microbial community in the tidal flat sediment. Within the sediment of the same treatments, the relative abundance of microbial OTUs was similar among the samples collected on any sampling date, indicating that the experimental treatments were reproduced.

We placed the N-treatment bottles 10 cm distant from the fish carcass since a part of the organic matter in the carcass may have been dissolved by bacterial activity and diffused into the surrounding environment through the water. However, the relative abundance of bacterial OTUs in the N-treatment did not differ significantly on any sampling date from those in the control treatment. This result implied that the ecological effects of the fish carcass on the bacterial assemblages were limited within a radius of 10 cm, suggesting that the dispersion of organic matter from the carcasses is spatially limited within a centimeter-scale in the tidal flat sediments. Since the carcasses contain energy and material with high nutritional quality, the organic matter may have been quickly consumed by bacteria before dispersing spatially. Alternatively, the low mobility of most bacteria may have limited the spatial translocation of these nutritional sources.

In the bacterial assemblages, the differences in the relative abundance of OTUs between the F-treatment and other treatments were relatively small at the start of the experiment but differed largely on day 9. This result implies that although bacteria can rapidly grow their populations, a unique bacterial community develops gradually in the sediment containing carcass. The result suggests that a time scale of several days is required for specific bacteria to colonize at a newly appeared environment by an animal carcass. However, we observed that the unique bacterial assemblages developed in the sediment vicinity of the fish carcass consisted mainly of bacterial taxa found in the ambient environment without any fish carcass. Indeed, we detected a nested structure in the OTU composition of bacterial assemblages in the F-treatment samples compared to the samples given the other treatments. This result supports our first hypothesis, i.e., that the bacterial assemblages developed in the sediment vicinity of a fish carcass consisted mainly of increases in the abundance of bacterial taxa that inhabited the ambient environment.

Pechal et al. (2013) [9] examined bacterial communities that developed in swine carcasses and showed that bacterial taxa using different organic matter was replaced in succession along with the progression of the decomposition. We did not examine whether the temporal changes in the OTU composition of the bacterial assemblages were related to compositional changes in the organic matter within the fish carcasses. However, we found that the OTU composition of the bacterial assemblages in the sediments with a fish carcass was gradually dissimilated from those in the ambient sediments, suggesting that successional changes in the composition of organic matter occurred and played a role in structuring the bacterial assemblages. The result supports our second hypothesis that a fish carcass develops unique bacteria assemblages in the vicinity sediments that differ compositionally from those in the sediments apparat from the fish carcass. Interestingly, the difference in the relative abundance of bacterial OTUs between the F-treatment and other treatments had become small by day 42, suggesting that it took >40 days for microbes to decompose the sardine carcass perfectly.

Like bacteria, ciliophorans are important organisms in the decomposition of dead organic matter [46, 47]. Nonetheless, few studies have examined ciliate assemblages associated with an animal carcass. This study revealed that the ciliophoran assemblages developed in the sediments close to the fish carcass were different from those in the ambient environment without the fish carcass. In the ciliophoran assemblages, the relative abundance of OTUs in the N-treatment but not the C-treatment did not differ significantly from that in the F-treatment samples, suggesting that the effects of the fish carcass on the ciliophorans extended spatially on a larger scale compared with the effects on the bacteria. These protozoans are mobile. Therefore, although it is speculative, some ciliophorans that grew in the F-treatment may have moved out and dispersed over a region with a radius greater than 10-cm, including an area where N-treatment bottles were placed. We also observed that in the ciliophoran assemblages, the β_sim_ values were relatively high among the samples regardless of the sampling date and treatments, indicating that their OTU composition was spatially and temporally more heterogeneous compared to the bacterial assemblages. Indeed, no nested structures were detected in the OTU composition of the F-treatment samples compared to those of other treatments except on day 9. Although it is not clear why the OTU composition of ciliophoran assemblages varied spatially and temporally, these organisms may be more sensitive to changes in the environmental conditions than bacteria.

As in the bacterial assemblages, the ciliophoran assemblages in the F-treatment differed largely from those in the other treatment on day 9, suggesting that these assemblages were compositionally related to each other. On day 9, the number of OTUs was small, and phylogenetically limited taxa such as Oligohymenophorea and Spirotrichea appeared predominately in the ciliophoran assemblages. When and at sites where bacterial decomposition activities are high, sediments may have become anoxic with the amplitude of hydrogen sulfide. Since some Oligohymenophorea and Spirotrichea species can grow under such conditions (e.g., [48]), they could colonize and maintain populations in which bacteria consume oxygen by decomposing the fish carcass. This possibility suggests that the F-treatment bottles harbored unique ciliate assemblages because of not only the increased bacteria abundance as a diet source but also environmental conditions created by the fish carcass with bacterial activities. To examine this possibility, we estimated what amount of variation in the similarity of the ciliophoran’s OTU composition among the samples was explained by the similarity of bacterial OTU composition and presence/absence of the fish carcass. The result showed that although only 4% of the variation was explained by the effects of the fish carcass in total, the similarity of bacterial assemblages explained 36% of the variation in the ciliophoran’s OTU composition. The results suggest that the ciliophoran’s OTU composition depends more directly on that of bacteria rather than indirectly on the presence of the fish carcass itself. This possibility supports our third hypothesis that a fish carcass modifies the environmental conditions in the surrounding sediments so that phylogenetically specific ciliophorans colonize through interactions with the bacterial assemblages. However, in this study, a large part of the variation in the ciliophoran’s composition was not explained by the bacterial assemblages, the fish carcass, or the sampling date.

In the bacterial assemblages, 29% of the variation in the similarity of OTU composition between samples was explained by the presence/absence of fish carcass. However, almost the same percentage of the variation was explained by the similarity of the ciliophoran’s OTU composition. Considering that most ciliophoran taxa are phagotrophic, this result suggests that prey-predator interactions between bacteria and ciliophorans may play a role in determining the shapes of these assemblages. The possibility implies that a unique but temporally limited food web is created in a spatially limited area around the carcass.

## Conclusion

The present study suggested that fish carcasses developed unique bacteria and ciliophoran assemblages in spatially limited areas in the tidal flat sediments and that the bacteria assemblages were formed by increases in the relative abundance of some taxa inhabiting the ambient environments, although, in the ciliophoran assemblages, some taxa that were rare in the ambient environment were often abundant. Our findings also suggested that these unique assemblages were created through not only the carcass itself but also biological interactions between bacteria and ciliophorans. Although we used sardine carcasses in this study, different microbial communities may develop on carcasses of different aquatic animals. In addition, factors such as temperature, the nutrient contents of sediments, and the presence or absence of macro-benthic animals are known to affect microbial communities in tidal flats [49–51]. Thus, to generalize the present findings, it is essential to determine how these factors affect the community structure of microbes in association with different types of fish carcasses.

## Supporting information

S1 Table Number of OTUs appeared in samples of each treatment. (DOCX)

S2 Table The top 10 numerically abundant OTUs of Bacteria in each sample in terms of the DNA read numbers. The OTU ID with the phylogenetic classification by SILVAngs is shown.
(XLSX)

S3 Table The top 10 numerically abundant OTUs of Ciliophora in each sample in terms of the DNA read numbers. The OTU ID with the phylogenetic classification by SILVAngs is shown.
(XLSX)

S4 Table Coefficients of determinations for regression models. These coefficients examine effects of sampling date, treatments, and ciliate or bacterial assemblages on the Horn similarity indices of bacterial and ciliate assemblages.
(DOCX)
