# Supplementary material for: Microbial communities developing within bulk sediments under fish carcasses on a tidal flat
Source: PLoS One. 2021 Feb 25;16(2):e0247220. doi: 10.1371/journal.pone.0247220 (PMC7906311; doi:10.1371/journal.pone.0247220)
Supplement: S1 Table — (DOCX) [file pone.0247220.s001.docx]

**Table S1**. Number of OTUs appeared in the samples of each treatment.

|  |  | Day 2 |  |  |  | Day 9 |  |  |  | Day 42 |  |  |
| --- | --- | --- | --- | --- | --- | --- | --- | --- | --- | --- | --- | --- |
|  |  | Sample number | |  |  | Sample number | |  |  | Sample number | |  |
| Organism | Treatment | 1 | 2 | 3 |  | 1 | 2 | 3 |  | 1 | 2 | 3 |
|  |  |  |  |  |  |  |  |  |  |  |  |  |
| Bacteria | F-treatment | 2918 | 3355 | 5487 |  | 611 | 2130 | 2213 |  | 3963 | 3027 | 2502 |
|  | N-treatment | 6113 | 6258 | 5743 |  | 5124 | 6264 | 6162 |  | 6526 | 6226 | 5717 |
|  | C-treatment | 6501 | 6529 |  |  | 6644 | 6503 |  |  | 6849 | 6790 |  |
|  |  |  |  |  |  |  |  |  |  |  |  |  |
| Ciliophora | F-treatment | 142 | 182 | 187 |  | 25 | 31 | 30 |  | 114 | 144 | 145 |
|  | N-treatment | 143 | 129 | 138 |  | 85 | 210 | 142 |  | 148 | 143 | 149 |
|  | C-treatment | 129 | 130 |  |  | 187 | 198 |  |  | 209 | 263 |  |
|  |  |  |  |  |  |  |  |  |  |  |  |  |
