# Supplementary material for: Microbial communities developing within bulk sediments under fish carcasses on a tidal flat
Source: PLoS One. 2021 Feb 25;16(2):e0247220. doi: 10.1371/journal.pone.0247220 (PMC7906311; doi:10.1371/journal.pone.0247220)
Supplement: S4 Table — These coefficients examine effects of sampling date, treatments, and ciliate or bacterial assemblages on the Horn similarity indices of bacterial and ciliate assemblages. (DOCX) [file pone.0247220.s004.docx]

**S4 Table**. Coefficients of determinations for the regression models examining effects of sampling date, treatments, and ciliophoran or bacteria assemblages on the Horn similarity indices of bacteria or ciliophoran assemblages.

| Objective variable | Explanatory variables | r^2^ | p |
| --- | --- | --- | --- |
|  |  |  |  |
| Bacteria | ciliate + date + fish | 0.569 | 0.0001 |
|  | ciliate + date | 0.401 | 0.0001 |
|  | ciliate + fish | 0.557 | 0.0001 |
|  | date + fish | 0.331 | 0.0001 |
|  | ciliate | 0.366 | 0.0001 |
|  | date | 0.006 | 0.0222 |
|  | fish | 0.319 | 0.0001 |
|  |  |  |  |
| Ciliophora | bacteria + date + fish | 0.505 | 0.0001 |
|  | bacteria + date | 0.497 | 0.0001 |
|  | bacteria + fish | 0.384 | 0.0001 |
|  | date + fish | 0.232 | 0.0001 |
|  | bacteria | 0.366 | 0.0001 |
|  | date | 0.166 | 0.0001 |
|  | fish | 0.053 | 0.0049 |
